# The Clinical Significance of CD169-Positive Lymph Node Macrophage in Patients with Breast Cancer

**DOI:** 10.1371/journal.pone.0166680

**Published:** 2016-11-18

**Authors:** Takuya Shiota, Yuko Miyasato, Koji Ohnishi, Mutsuko Yamamoto-Ibusuki, Yutaka Yamamoto, Hirotaka Iwase, Motohiro Takeya, Yoshihiro Komohara

**Affiliations:** 1 Department of Cell Pathology Graduate School of Medical Sciences, Kumamoto University, Kumamoto, Japan; 2 Department of Molecular-Targeting Therapy for Breast Cancer, Kumamoto University Hospital, Kumamoto, Japan; 3 Department of Breast and Endocrine Surgery, Graduate School of Medical Science, Kumamoto University, Kumamoto, Japan; Istituto Superiore di Sanità, ITALY

## Abstract

The immune status of patients can impact on the clinical course of cancer. Lymph node (LN) macrophages play critical roles in anti-cancer immunity via the activation of cytotoxic T-lymphocytes (CTLs). In this study, the prognostic significance of CD169^+^ LN macrophages was examined in patients with breast cancer. For this purpose the number of CD169^+^ cells and their ratio relative to total macrophages (CD68^+^) in regional LNs (RLNs), as well as the number of CD8^+^ CTLs in tumor tissues, were investigated using immunohistochemistry of paraffin-embedded tissue samples from 146 patients with breast cancer. The association of these data with clinicopathological factors was then analyzed. The number of cells positive for the pan-macrophage marker CD68 remained relatively uniform, while the number of CD169^+^ cells varied across all cases. Moreover, a high density of CD169^+^ cells correlated with early clinical stage and no LN metastasis, while a higher CD169^+^ to CD68^+^ ratio was significantly associated with small tumor size and a low Ki-67^+^ rate. There was also a significant correlation between the number of CD8^+^ CTLs and that of CD169^+^ macrophages in high grade breast cancer cases with a Ki-67 index greater than 40%. However, neither the density nor the ratio of CD169^+^ cells, nor the density of CD8^+^ CTLs, were associated with relapse-free survival, distant relapse-free survival, or breast cancer-specific survival. These findings suggest that CD169^+^ macrophages in RLNs might be a useful marker for assessing clinical stage, including LN states, in patients with breast cancer.

## Introduction

Breast cancer is a frequent malignant tumor that occurs predominantly in women. The number of patients affected by this disease has continuously increased in recent years. It is well established that breast cancer cell metastasis to the axillary lymph node is associated with increased mortality [1, 2]. Notably, the immune status of patients can influence its clinical course [3]. For instance, decreased infiltration of CD8^+^ cytotoxic T-lymphocytes (CTLs) in tumor has been associated with lymph node metastasis and poor response to neoadjuvant chemotherapy [4–6], while the activation of immune responses has been related to improved overall survival in patients with breast cancer [7]. Thus, an anti-cancer immune status is thought to be important for preventing cancer recurrence and metastasis. However, the mechanisms related to the induction or maintenance of anti-cancer immune reactions are not fully understood.

Macrophages play an important role in the immune system. Their functions include phagocytosis, cytokine secretion, and antigen presentation. It is widely accepted that classically activated (M1) macrophages preferentially contribute to the activation of Th1 type immune reactions, while alternatively activated (M2) macrophages are associated with Th2 type immune reactions or tolerance [8, 9].

**Fig 1:**
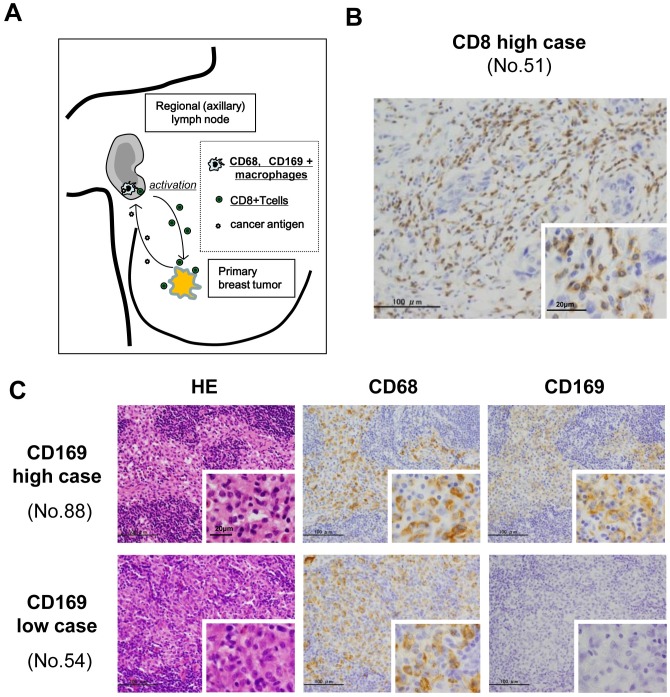
Representative immunostaining of CD8, CD68, and CD169. Tumor infiltrating CD8^+^ lymphocytes were evaluated in primary cancer tissues, while CD68^+^ and CD169^+^ sinus macrophages were evaluated in RLNs (A). Representative images of CD8 (B) and of CD68 and CD169 (C) immunostaining. HE, Hematoxylin-Eosin staining.

Recent findings indicate that the immune balance in the tumor-draining regional lymph node (RLN) is significantly involved in anti-cancer immune responses. Moreover, phenotypic change related to the Th1/Th2 immune balance in the LN has been related to clinical course in patients with breast cancer or melanoma [10, 11]. CD169^+^ sinus macrophages have been shown to activate CTLs by presentation of dead cell-associated cancer antigens in LNs (Fig 1A); in contrast, their depletion abrogates the establishment of an anti-cancer immune response [12]. Thus, sinus macrophages in RLNs are believed to play an important role in anti-cancer immune responses [13, 14].

CD169, also known as Sialoadhesin or sialic acid-binding immunoglobulin-like lectin (Siglec)-1, is a cell surface marker on macrophages that belongs to the Siglec family and that functions as a sialic acid receptor. CD169 is expressed on macrophages of secondary lymphoid tissues such as those of the lymph node or spleen [15]. More specifically, these CD169^+^ macrophages are found at the marginal zone of the spleen and at the subcapsular sinus of the lymph node. At these sites, CD169^+^ macrophages are readily able to encounter pathogens and present antigens to immune cells [16, 17]. CD169 is considered to be a marker of M1 macrophages because it is upregulated in human macrophages by stimulation with interferons [18].

Based on the above information, we speculated that CD169^+^ macrophages in RLNs might play a role in the establishment and maintenance of anti-cancer immunity. We therefore evaluated the relationships between CD169^+^ macrophages in RLNs and clinicopathological factors in patients with breast cancer.

## Materials and Methods

### Patients

Paraffin-embedded tumor and matched RLN samples were prepared from 167 patients who were diagnosed with ductal carcinoma (21 with ductal carcinoma in situ and 146 with invasive ductal carcinoma) between 2001–2008 at Kumamoto University Hospital. For the analysis of CD8^+^ lymphocytes in primary lesions, 136 patient samples were examined. The remaining 10 samples were unsuitable for evaluation. With respect to clinicopathological factors, estrogen receptor (ER) values were missing for 2 patients, while Ki-67 data were missing for 5 out of the 146 patient RLN samples and for 4 out of 136 patient tumor samples. Patients were not treated with neoadjuvant chemotherapy. After surgery, patients were followed for a median period of 86 months (range 1–159 months). Axillary lymph nodes, including sentinel and non-sentinel lymph nodes, were used as RLNs. In cases with LN metastasis, only cancer cell-free LNs were used for the analysis. All samples were obtained with informed patient consent in accordance with protocols approved by the Human Ethics Review Committee of Kumamoto University (No.1176). The participants provide their written informed consent to participate in this study. The present study data are managed and protected by the Department of Breast and Endocrine Surgery, Graduate School of Medical Science, Kumamoto University. Tissue samples were fixed in 10% neutral buffered formalin and were embedded in paraffin as per routine methodology. Patient characteristics are indicated in S1 Table.

### Immunostaining and cell counting

Immunohistochemical staining was performed on 3 μm-thick sections obtained from paraffin-embedded tissues. Mouse monoclonal antibodies against CD68 (clone PG-M1; Dako, Glostrup, Denmark), CD8 (clone C8/144B; Nichirei, Tokyo, Japan) and CD169 (clone HSn7D2; Santa Cruz Biotechnology, CA) were used. After the samples were reacted with primary antibodies, they were incubated with horseradish peroxidase-labeled secondary anti-mouse antibody (Nichirei, Tokyo, Japan). Signals were visualized using the diaminobenzidine system (Nichirei, Tokyo, Japan). Normal mouse immunoglobulin (DAKO, Glostrup, Denmark) was used as a negative control and no signal was observed in those control sections. Data regarding the Ki-67 labeling index, as well as the statuses of ER, PgR, and Her2, were previously evaluated by our research group [19, 20]. CD68-, CD169-, and CD8-positive staining was counted in four randomly selected fields-of-view by two pathologists (TS and YM) who were blind to patient information. The average numbers of counted cells were determined and were used to calculate the number per mm^2^. To evaluate lymph node metastasis, sinus macrophages were counted in non-metastatic regions. The detailed methods of cell counting in RLNs have been previously described [21].

### Statistical analysis

JMP10 (SAS Institute, Chicago, IL, USA) was used for statistical analysis. The cumulative survival rate was compared between two groups using a log-rank test and was generalized using the Wilcoxon test. The median was used as the cutoff value for the comparison of two groups. Since the data were non-parametric the Mann-Whitney U and Kruskal-Wallis tests were used. A p-value of <0.05 was considered to represent a statistically significant difference.

## Results

### A high density of CD169^+^ macrophages is associated with small tumor size, no lymph node metastasis, early clinical stage, and a low Ki-67 index

**Fig 2:**
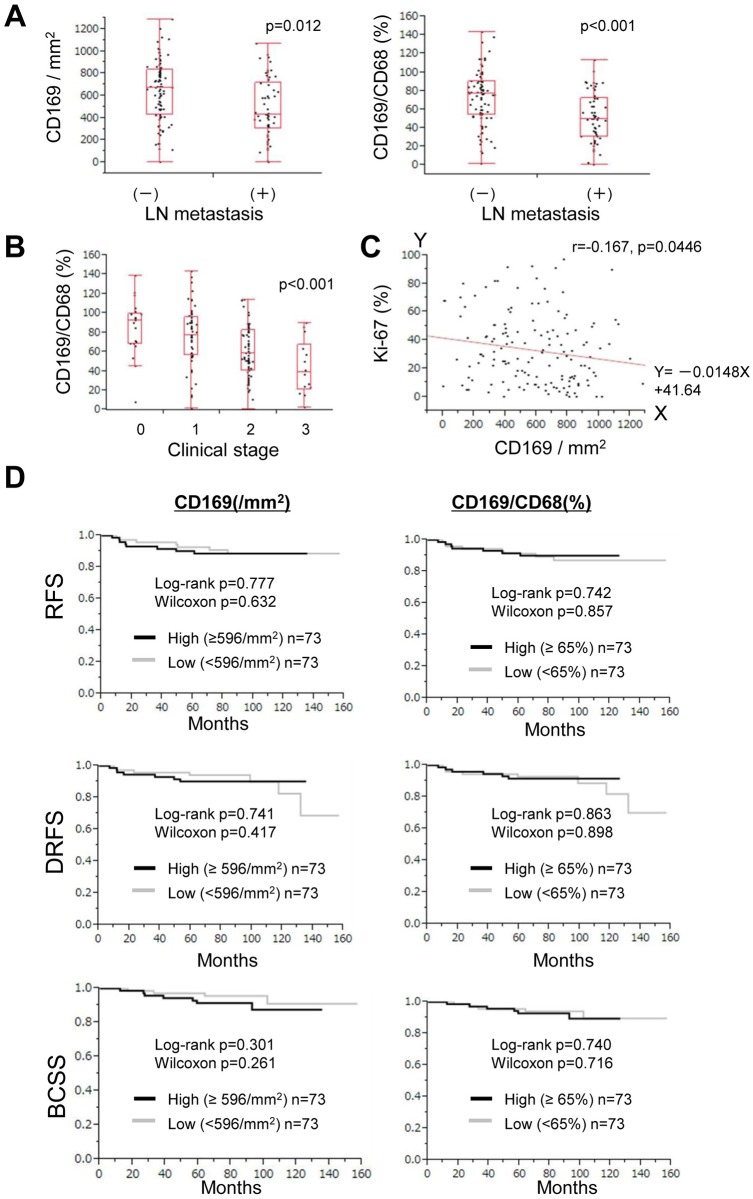
Statistical analysis of associations of sinus macrophages with clinicopathological factors. Analysis of the association of CD169^+^ macrophage density and the CD169^+^/CD68^+^ macrophage ratio with LN metastasis (A), clinical stage (B), the Ki-67 index (C) and prognosis (D). The Mann-Whitney U test and Kruskal-Wallis tests were performed to examine the prognostic value of CD169 in (D).

To investigate the prognostic value of CD169^+^ macrophages in breast cancer, we first compared their density in RLNs to that of CD8^+^ CTLs in resected primary tumors (Fig 1A) of paired patient samples, and then determined their correlation with clinicopathological features. Immunostaining of CD8^+^ lymphocytes is shown in Fig 1B and S1A Fig, while that of CD68^+^ and CD169^+^ macrophages is shown in Fig 1C, and S1A Fig The average density of CD68^+^ pan-macrophages in the RLN sinus area was 909.56 cells/mm^2^ (n = 146, range = 484.27–1397.67, median = 904.22) (S1B Fig). There were no significant differences in the density of CD68^+^ macrophages across cases or in their correlation with clinicopathological factors (Fig 1C, Table 1). In contrast, the density of CD169^+^ macrophages in the RLN sinus area, as well as their ratio relative to CD68^+^ macrophages, did vary across cases (n = 146, number of cells/mm^2^ (ratio): average = 585.05 (64.78), range = 1.00–1282.55 (0.40–143.36), median = 596.83 (65.37)) (Fig 1C, and S1B Fig). When the density of CD169^+^ macrophages was examined in cases with and without LN metastasis, it was significantly higher in cases without LN metastasis than in those with LN metastasis; the average density was 643.61 cells/mm^2^ (range = 1.00–1282.55) and 485.09 cells/mm^2^ (range = 3.78–1066.9), respectively (p = 0.0012). Additionally, the ratio of CD169^+^ macrophages relative to total macrophage numbers, as represented by CD68^+^ cells, was significantly higher in samples without LN metastasis (72.3, range = 0.94–143.36) than in those with LN metastasis (51.95, range = 0.4–112.93) (p<0.0001) (Table 1, Fig 2A). A greater number and higher ratio of CD169^+^ macrophages were also associated with small tumors of <2 cm in size (n = 62; number of cells/mm^2^ (ratio): average = 642.86 (73.76), range = 1.00–1282.55 (0.40–143.36), median = 678.17 (77.10)) rather than with tumors that were ≥2 cm in size (n = 84, number of cells/mm^2^ (ratio): average = 542.37 (58.15), range = 11.35–1109.06 (1.98–113.73), median = 544.80 (58.60)) (number, p = 0.029; ratio, p = 0.002) (Table 1). Moreover, the ratio of CD169^+^ to CD68^+^ macrophages was negatively correlated with clinical stage (p<0.001) (Fig 2B). Additionally, the number of CD169^+^ cells and their ratio to CD68^+^ macrophages were negatively correlated with the Ki-67 index (p = 0.0446) (Table 1, Fig 2C). No significant correlations were detected between the density of CD169^+^ cells or the CD169^+^/CD68^+^ macrophage ratio and clinical prognoses such as relapse-free survival (RFS), distant relapse-free survival (DRFS), or breast cancer-specific survival (BCSS) (Fig 2D, Tables 2–4).

**Table 1:** Association of CD169^+^, CD68^+^, and CD8^+^expressing cells with clinicopathological parameters in the invasive breast cancer cohort.

Clinical Parameters		CD68(/mm2)		CD169(/mm2)		CD169/CD68(%)			CD8(/mm2)	
	N	Mean	p value	Mean	p value	Mean	p value	N	Mean	p value
**Age**										
**<55**	**70**	**915.66**	**0.80**	**555.89**	**0.24**	**61.61**	**0.21**	**64**	**173.89**	**0.68**
**≧56 (=median)**	**76**	**903.94**		**611.90**		**67.69**		**72**	**186.11**	
**Menopause**										
**Pre-**	**46**	**912.12**	**0.95**	**579.46**	**0.79**	**63.65**	**0.70**	**93**	**183.66**	**0.80**
**Post-**	**100**	**908.38**		**587.62**		**65.30**		**43**	**173.21**	
**Tumor size**										
**<2cm**	**62**	**888.78**	**0.20**	**642.86**	**0.029**	**73.76**	**0.002**	**58**	**165.06**	**0.58**
**≧2cm**	**84**	**924.90**		**542.37**		**58.15**		**78**	**191.74**	
**Clinical stage**										
**Stage1**	**57**	**875.00**	**0.074**	**660.00**	**0.009**	**76.40**	**<0.001**	**52**	**160.86**	**0.31**
**Stage2,3**	**89**	**932.00**		**537.00**		**57.40**		**84**	**192.43**	
**Nodal status**										
**Negative**	**92**	**903.07**	**0.56**	**643.71**	**0.001**	**72.30**	**<0.001**	**85**	**171.08**	**0.43**
**Positive**	**54**	**920.62**		**485.09**		**51.95**		**51**	**195.82**	
**Nuclear grade**										
**Grade 1**	**71**	**901.65**	**0.58**	**616.12**	**0.19**	**69.32**	**0.083**	**65**	**160.78**	**0.053**
**Grade 2,3**	**75**	**917.05**		**555.63**		**60.48**		**71**	**198.28**	
**ERα**										
**negative**	**37**	**935.68**	**0.40**	**544.57**	**0.25**	**58.45**	**0.15**	**34**	**209.34**	**0.20**
**positive**	**107**	**896.80**		**596.44**		**66.96**		**100**	**168.60**	
**PgR**										
**negative**	**51**	**909.63**	**0.92**	**542.64**	**0.15**	**59.66**	**0.12**	**47**	**215.07**	**0.053**
**positive**	**95**	**909.52**		**607.81**		**67.52**		**89**	**162.03**	
**HER2**										
**negative**	**116**	**906.70**	**0.82**	**598.98**	**0.20**	**66.65**	**0.12**	**111**	**172.25**	**0.14**
**positive**	**30**	**920.62**		**531.18**		**57.52**		**25**	**216.38**	
**Ki67**										
**<20%**	**48**	**875.10**	**0.13**	**625.80**	**0.083**	**72.52**	**0.005**	**44**	**139.63**	**0.023**
**≧20%**	**93**	**932.19**		**551.76**		**58.90**		**88**	**198.16**	

**Table 2:** Univariate and multivariate analysis of factors for relapse-free survival.

Variables		Reference	Univariate analysis				Multivariate analysis			
			p value	HR	95% CI		p value	HR	95% CI	
					Lower	Upper			Lower	Upper
**Menopause**	**pre vs. post**	**post**	**0.22**	**1.91**	**0.67**	**5.33**				
**Tumor size**	**≦2cm vs. >2cm**	**≦2cm**	**0.17**	**2.14**	**0.73**	**7.74**	**0.35**	**2.63**	**0.39**	**25.3**
**Clinical stage**	**1 vs 2, 3**	**1**	**0.31**	**1.77**	**0.61**	**6.4**	**0.77**	**0.72**	**0.091**	**5.98**
**Nodal status**	**positive vs. negative**	**negative**	**0.19**	**1.96**	**0.71**	**5.6**				
**Nuclear Grade**	**1 vs 2, 3**	**1**	**0.21**	**1.96**	**0.7**	**6.31**				
**ERα**	**negative vs. positive**	**positive**	**0.0017**	**5.23**	**1.88**	**15.6**	**0.0054**	**9.41**	**1.91**	**48.2**
**PgR**	**negative vs. positive**	**positive**	**0.11**	**2.28**	**0.82**	**6.51**	**0.32**	**0.44**	**0.085**	**2.22**
**Her2**	**positive vs. negative**	**negative**	**0.59**	**1.38**	**0.38**	**4.04**	**0.45**	**0.60**	**0.14**	**2.24**
**Ki67**	**> 20% vs. ≦20%**	**≦20%**	**0.049**	**3.63**	**1**	**23.2**	**0.40**	**1.95**	**0.44**	**13.6**
**CD68**	**low vs. high**	**low**	**0.76**	**1.17**	**0.42**	**3.35**				
**CD169**	**low vs. high**	**high**	**0.78**	**0.86**	**0.3**	**2.41**	**0.54**	**0.57**	**0.11**	**3.64**
**CD169/CD68**	**low vs. high**	**high**	**0.74**	**1.18**	**0.43**	**3.38**	**0.68**	**1.46**	**0.22**	**7.84**
**CD8**	**low vs. high**	**high**	**0.86**	**0.92**	**0.32**	**2.55**	**0.91**	**0.94**	**0.31**	**2.78**

**Table 3:** Univariate and multivariate analysis of factors for distant relapse-free survival.

Variables		Reference	Univariate analysis				Multivariate analysis			
			p value	HR	95% CI		p value	HR	95% CI	
					Lower	Upper			Lower	Upper
**Menopause**	**pre vs. post**	**post**	**0.76**	**1.19**	**0.37**	**3.45**				
**Tumor size**	**≦2cm vs. >2cm**	**≦2cm**	**0.15**	**2.41**	**0.74**	**10.8**	**0.62**	**1.80**	**0.21**	**23.3**
**Clinical stage**	**1 vs 2, 3**	**1**	**0.31**	**1.89**	**0.57**	**8.52**	**0.81**	**1.33**	**0.14**	**15.6**
**Nodal status**	**positive vs. negative**	**negative**	**0.16**	**2.14**	**0.74**	**6.54**				
**Nuclear Grade**	**1 vs 2, 3**	**1**	**0.4**	**1.59**	**0.54**	**5.2**				
**ERα**	**negative vs. positive**	**positive**	**0.0003**	**7.43**	**2.46**	**27.3**	**0.0034**	**9.29**	**2.06**	**48.2**
**PgR**	**negative vs. positive**	**positive**	**0.014**	**4.06**	**1.32**	**15.1**	**0.86**	**1.16**	**0.23**	**6.12**
**Her2**	**positive vs. negative**	**negative**	**0.75**	**1.22**	**0.33**	**3.72**	**0.083**	**0.30**	**0.063**	**1.17**
**Ki67**	**> 20% vs. ≦20%**	**≦20%**	**0.027**	**6.05**	**1.18**	**110.5**	**0.29**	**2.77**	**0.47**	**52.8**
**CD68**	**low vs. high**	**low**	**0.74**	**0.82**	**0.41**	**3.66**				
**CD169**	**low vs. high**	**high**	**0.74**	**0.84**	**0.28**	**2.48**	**0.63**	**0.61**	**0.096**	**5.25**
**CD169/CD68**	**low vs. high**	**high**	**0.86**	**1.1**	**0.36**	**3.44**	**1.00**	**0.99**	**0.10**	**6.80**
**CD8**	**low vs. high**	**high**	**0.93**	**1.05**	**0.36**	**3.08**	**0.77**	**1.18**	**0.38**	**3.75**

**Table 4:** Univariate and multivariate analysis of factors for breast cancer-specific survival.

Variables		Reference	Univariate analysis				Multivariate analysis			
			p value	HR	95% CI		p value	HR	95% CI	
					Lower	Upper			Lower	Upper
**Menopause**	**pre vs. post**	**post**	**0.79**	**1.18**	**0.31**	**3.92**				
**Tumor size**	**≦2cm vs. >2cm**	**≦2cm**	**0.11**	**3.14**	**0.8**	**20.7**	**0.081**	**15.1**	**0.74**	**438.9**
**Clinical stage**	**1 vs 2, 3**	**1**	**0.53**	**1.52**	**0.43**	**6.99**	**0.32**	**0.23**	**0.015**	**4.18**
**Nodal status**	**positive vs. negative**	**negative**	**0.32**	**1.84**	**0.55**	**6.4**				
**Nuclear Grade**	**1 vs 2, 3**	**1**	**0.16**	**2.47**	**0.71**	**11.3**				
**ERα**	**negative vs. positive**	**positive**	**<0.001**	**14**	**3.61**	**92.2**	**0.0012**	**18.7**	**2.97**	**172.9**
**PgR**	**negative vs. positive**	**positive**	**0.013**	**4.78**	**1.37**	**21.9**	**0.95**	**0.94**	**0.16**	**6.57**
**Her2**	**positive vs. negative**	**negative**	**0.78**	**1.22**	**0.26**	**4.24**	**0.22**	**0.38**	**0.069**	**1.77**
**Ki67**	**> 20% vs. ≦20%**	**≦20%**	**0.054**	**0.97**	**0.41**	**94**	**0.72**	**1.50**	**0.20**	**30.6**
**CD68**	**low vs. high**	**low**	**0.73**	**0.81**	**0.23**	**2.71**				
**CD169**	**low vs. high**	**high**	**0.3**	**0.53**	**0.14**	**1.75**	**0.55**	**0.49**	**0.060**	**5.55**
**CD169/CD68**	**low vs. high**	**high**	**0.74**	**0.82**	**0.24**	**2.72**	**0.75**	**0.68**	**0.048**	**6.07**
**CD8**	**low vs. high**	**high**	**0.82**	**0.87**	**0.25**	**2.89**	**0.75**	**0.80**	**0.19**	**3.09**

### The density of CD8^+^ lymphocytes is not associated with clinicopathological factors or RLN macrophages

**Fig 3:**
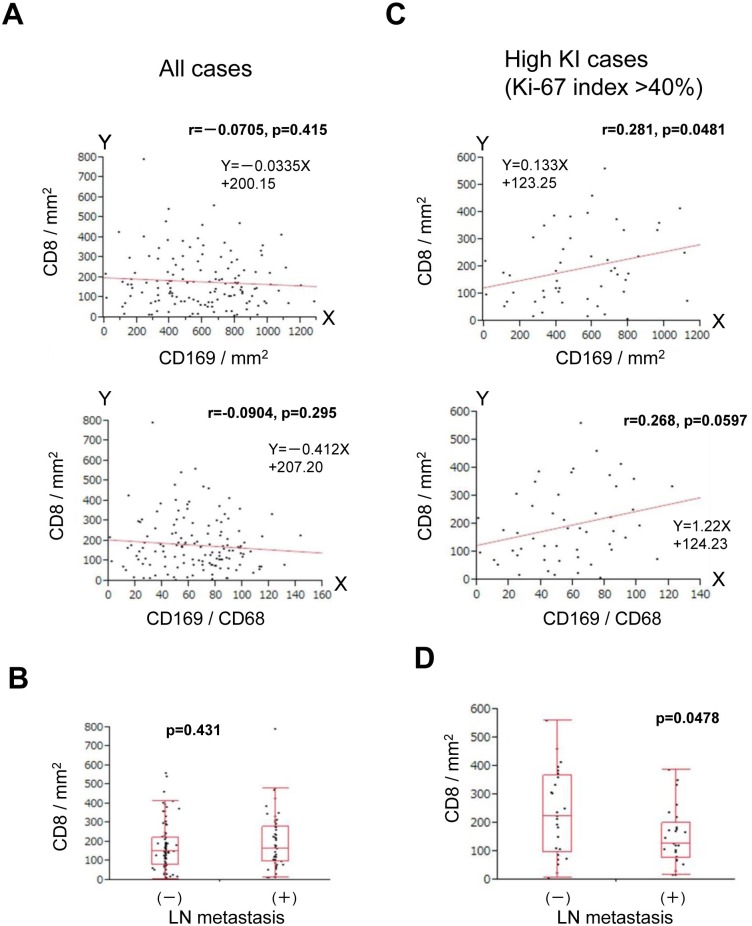
Statistical analysis of associations of CD8^+^ lymphocytes with CD169^+^ macrophages and clinicopathological factors. Analysis of the association of CD8^+^ lymphocyte density with CD169^+^ macrophage density (upper) and the CD169^+^/CD68^+^ macrophage ratio (lower) (A) and with LN metastasis (B) in all cases. Similar analyses to (A) and (B) that were performed in cases with a high Ki-67 index (>40%) are shown in (C) and (D), respectively.

Since the infiltration of CD8+ lymphocytes in tumor tissue indicates an anti-cancer immune response, we determined CD8+ cell density in tumors, compared it to that of CD169^+^ macrophages in matched RLN tissues, and further determined its correlation with clinicopathological factors. Based on previous studies, we expected that the CD169^+^ macrophage cell density, or the CD169^+^/CD68^+^ macrophage ratio, would be associated with the density of CD8^+^ lymphocytes; however, no significant correlations were detected (density, p = 0.415; ratio, p = 0.295) (Fig 3A). There were also no significant correlations between the density of CD8^+^ lymphocytes and clinicopathological factors (Table 1, Fig 3B, and S2 Fig).

### A high density of CD8^+^ lymphocytes is associated with a high density of CD169^+^ macrophages and a low incidence of LN metastasis in high grade breast cancer cases

Next, we evaluated the relationships between CD169^+^ macrophages and CD8^+^ lymphocytes in cases with higher malignant potential, as indicated by a Ki-67 index of >40%. Interestingly, analysis of these cases indicated that a high density of CD8^+^ lymphocytes was associated with a high density of CD169^+^ macrophages (p = 0.0481) (Fig 3C). When the association of CD8^+^ lymphocyte density with LN metastasis was considered in these Ki-67-high cases, a higher density of CD8^+^ lymphocytes was observed in cases without LN metastasis (n = 25, average = 235.08, range = 6.86–559.58, median = 224.89) than in those with LN metastasis (n = 25, average = 152.43, range = 15.28–385.37, median = 126.64) (p = 0.0478) (Fig 3D).

## Discussion

We demonstrated that the CD169^+^ cell density and the CD169^+^/CD68^+^ macrophage ratio in the RLNs of breast cancer patients correlated with early clinical stage and no LN metastasis as well as with an increased density of CD8^+^ lymphocytes in cases with a Ki-67 index of >40%. These findings are in line with a previous study that showed that CD169^+^ cells in RLNs are closely associated with the priming of antigen-specific CTLs [12]. Dendritic cells are thought to be the most professional antigen-presenting cells in LNs. Recently, however, Benhard et al. reported that sinus CD169^+^ macrophages induce antigen-specific CTLs in the context of dendritic cell depletion [22]. Our previous studies also demonstrated significant associations between CD169^+^ cells in RLNs and CD8^+^ CTL infiltration in primary cancer tissues derived from colorectal cancer and malignant melanoma. Moreover, we reported that patients with a higher CD169^+^ cell number or higher CD169^+^/CD68^+^ macrophage ratio in RLNs had a better clinical prognosis than those with lower levels of CD169^+^ cells in those cancers [18, 21]. In the present study, significant correlations were detected between CD169^+^ cells in RLNs and CD8^+^ CTL infiltration in primary cancer tissues only in cases with a Ki-67 index of >40%. Since Ki-67 is a marker of cell proliferation, these data suggest that CD169^+^ cells in RLNs might be specifically involved in the priming of CD8^+^ CTLs in breast cancers with higher malignant potential.

CD169^+^ macrophages might also contribute to anti-cancer immunity through additional mechanisms. Regulatory T cells (Treg) express CD169 ligands on their surface that allow their interaction with CD169^+^ macrophages, which results in inflammation by CD169^+^ macrophage-mediated Treg suppression [23]. CD169^+^ macrophages have also been demonstrated to uptake and present lipid antigens that can lead to *i*NKT cell activation [24]. Thus, CD169^+^ macrophages might influence Treg or *i*NKT cells in RLNs to thereby impact on anti-cancer immunity. Regarding the exact mechanism by which macrophage CD169 is activated, we have previously shown that CD169 expression is significantly upregulated by type 1 interferon (IFN), suggesting that its expression on sinus macrophages could be influenced by IFN-a secreting infiltrating macrophages and plasmacytoid dendritic cells in RLNs [21]. Further studies related to Treg and *i*NKT cells are necessary to uncover the detailed mechanisms that underlie the induction of anti-cancer immune responses by CD169^+^ macrophages in breast cancer.

A high density of CD8^+^ CTLs shows a good correlation with better clinical prognosis in several malignant tumors such as colorectal cancer and bladder cancer [25, 26]. In breast cancer, a higher density of either CD3^+^ or CD8^+^ lymphocytes predicted better response to neoadjuvant chemotherapy [27, 28]. Furthermore, CD8^+^ CTL infiltration in primary breast cancer suppressed brain metastasis [29]. However, few reports have shown a significant correlation between CD8^+^ lymphocytes and clinical prognosis. In one such previous study, Menard et al. reported that a high number of infiltrating CD8^+^ lymphocytes in breast cancer was associated with better prognosis in patients younger than 40 years old but not in those older than 40 years old [30]. Although it would have been interesting to evaluate the prognostic value of CD169^+^ cells in RLNs and CD8^+^ CTL infiltration in primary cancer tissues using this age cut-off, our dataset only had a few cases where patients were younger than 40 years old (n = 9) and therefore statistical analysis using such an age cut-off was not performed. In another study, Liu et al. demonstrated that CD8^+^ tumor infiltrating lymphocytes were an independent prognostic factor associated with better patient survival in basal-like triple negative breast cancer (TNBC) [31]. Indeed, TNBCs are associated with an increased cytotoxic T-cell-mediated immune response, although the presence of programmed cell death receptor ligand 1, an immune checkpoint molecule, is able to suppress T-cell activation [32]. The current dataset had only a few cases of TNBC (n = 17), and therefore analysis of this subset was not performed. However, it would be of interest in future studies to evaluate the statistical correlation between CD169^+^ cells in RLNs and CD8^+^ CTL infiltration in TNBCs.

When we focused on cases with a high Ki67 index of >40%, cases with a high density of CD8^+^ T cells showed a tendency to have a better prognosis than those with a low CD8^+^ T cell density (S2A and S2B Fig). In future studies, investigation of a larger cohort of patients with a high Ki67 index would help to more fully comprehend the relationships between CD8+ T cell infiltration and clinical prognosis. In the present study, we were unable to determine why the CD169^+^ cell density and the CD169^+^/CD68^+^ macrophage ratio in RLNs correlated with clinical stage and LN metastasis. However, many other studies that used animal models have suggested that CD169^+^ macrophages are significantly involved in anti-cancer immune responses, and therefore such responses may be involved in these correlations.

Immunotherapy is of interest as a new anti-cancer therapy; however, there are few biomarkers for evaluating anti-cancer immune responses. Many studies are now being carried out in an attempt to develop methods by which anti-cancer immune responses can be evaluated in order to predict the anti-cancer effect of immune-checkpoint inhibitors. While further work is required, our study suggests that the CD169^+^ cell density and the proportion of CD169^+^ to CD68^+^ macrophages in RLNs might be useful for examination of the status of anti-cancer immune responses. Moreover, these parameters might also represent a useful marker for assessment of clinical stage including LN status in patients with breast cancer.

## Supporting Information

S1 Fig Images of immunostaining of CD8 and CD169 in other cases (scale bar, 100 μm) (A). Scatter diagrams for CD68 and CD169 (B).
(TIF)

S2 Fig Statistical analysis of the associations of CD8^+^ lymphocytes with clinical prognosis in all cases (A) and in cases with a high Ki-67 index (>40%) (B). (TIF)

S1 Table Patient characteristics. (DOCX)
