# Supplementary material for: The Clinical Significance of CD169-Positive Lymph Node Macrophage in Patients with Breast Cancer
Source: PLoS One. 2016 Nov 18;11(11):e0166680. doi: 10.1371/journal.pone.0166680 (PMC5115774; doi:10.1371/journal.pone.0166680)
Supplement: S1 Table — (DOCX) [file pone.0166680.s003.docx]

**S1 Table Patient characteristics**

| **Characteristics** | **n(total=146)** | **%** |
| --- | --- | --- |
| **Age(years)** |  |  |
| **Median** | **56** |  |
| **Range** | **27-93** |  |
| **Histological subtype** |  |  |
| **Papillotubular** | **100** | **68.5** |
| **Solid-tubular** | **21** | **14.4** |
| **Scirrhous** | **25** | **17.1** |
| **Histological grade** |  |  |
| **1** | **50** | **34.3** |
| **2** | **65** | **44.5** |
| **3** | **31** | **21.2** |
| **Clinical stage** |  |  |
| **Ⅰ** | **58** | **39.7** |
| **ⅡA** | **47** | **32.2** |
| **ⅡB** | **28** | **19.2** |
| **ⅢA/B** | **13** | **8.9** |
| **Lymph node metastasis** |  |  |
| **Positive** | **54** | **37** |
| **Negative** | **92** | **63** |
| **Adjuvant therapy** |  |  |
| **Yes** | **119** | **81.5** |
| **No** | **26** | **17.8** |
| **Unknown** | **1** | **0.7** |
